# A FRACTIONAL ORDER HIV/AIDS MODEL USING CAPUTO-FABRIZIO OPERATOR

**DOI:** 10.21010/ajidv15i2S.1

**Published:** 2021-09-01

**Authors:** Ebenezar Nkemjika Unaegbu, Ifeanyi Sunday Onah, Moses Oladotun Oyesanya

**Affiliations:** * Department of Mathematics, Salem University, Lokojaia; ** Department of Mathematics, University of Nigeria, Nsukka, Nigeria; *** Department of Mathematics, University of Nigeria, Nsukka, Nigeria

**Keywords:** Mathematical models, HIV/AIDS, Caputo Fabrizio derivative, Existence, Uniqueness and stability, Laplace transform, Numerical simulation

## Abstract

**Background:**

HIV is a virus that is directed at destroying the human immune system thereby exposing the human body to the risk of been affected by other common illnesses and if it is not treated, it generates a more chronic illness called AIDS.

**Materials and Methods:**

In this paper, we employed the fixed-point theory in developing the uniqueness and existence of a solution of fractional order HIV/AIDS model having Caputo-Fabrizio operator. This approach adopted in this work is not conventional when solving biological models by fractional derivatives.

**Results:**

The results showed that the model has two equilibrium points namely, disease-free, and endemic equilibrium points, respectively. We showed conditions necessitating the existence of the endemic equilibrium point and showed that the disease-free equilibrium point is locally asymptotically stable. We also tested the stability of our solution using the iterative Laplace transform method on our model which was also shown stable agreeing with the disease-free equilibrium.

**Conclusions:**

Numerical simulations of our model showed clear comparison with our analytical results. The numerical solutions show that given fractional operator like the Caputo-Fabrizio operator, it is less noisy and hence plays a major role in making a precise decision and gives room or opportunity (‘freedom’) to use data of specific patients as the model can be easily adjusted to accommodate this, as it a better fit for the patients’ data and provide meaningful predictions. Finally, the result showed the advantage of using fractional order derivative in the analysis of the dynamics of HIV/AIDS over the classical case.

## Introduction

Human Immune Deficiency Virus (HIV) is a lentivirus that attacks cells in the human immune system. It is known that the immune system is a shield in our body that is aimed at defending man from been easily affected by illness. The virus on entrance into the body system damages the white blood cell (T-helper cell), and replicates itself inside the white blood cells, making it difficult for it to be destroyed. This leads to the reduction of the T-helper cells which recognizes infected cells and thereby making room for the virus to cause permanent damage to the immune system. HIV can be transmitted through body fluid that includes (i) blood (ii) breast milk (iii) semen (iv) vagina and (v) rectal fluids (Cai et al., 2009). The number of viruses in human blood is what determines the stage of the disease. Most times, these stages are meant to conform to the CD4+ T-cell count ranges. In a complete person, the CD4+ counts are between 500 to 1500 per cubic millimeter and once this count is 200 per cubic millimeter or less than that, there is a high degree likelihood that the HIV patient is said to have fully developed into AIDS (Stoddart and Reyes, 2006). AIDS has now become a global epidemic since the first incidence case as reported in 1981 (Abdo et al., 2020). Reports show that over 75 million people in the world are now living with HIV infection, another 37.9 million people are currently been infected and another 32 million people all over the world have been reported dead due to AIDS as of 2018 (Bushnaq et al., 2018; Taneco-Hernandez et al., 2020). If an individual infected with HIV remains untreated, such person’s progress to AIDS within a decade from its infection period and the life expectancy is about three years after been diagnosed. This is not a lifeline, because some individuals tend to develop several opportunistic illnesses and such as limiting their survival within this period. However, treatment with antiretroviral can prevent AIDS from developing. In fact, worldwide, it is estimated that 3-4 million death were prevented in 2018 as there was an improved medication assisted treatment (MAT) to HIV/AIDS (WHO/UNAIDS, 2005).

A mathematical model is a variable scientific tool that has been identified and used in studying and analyzing the transmission dynamics of infectious diseases and has been proven to be successful (Collins and Dully, 2018; Agusto, 2009; Joshi, 2002; Collins and Dully, 2016; Taneco-Hernandez and Vargas-De-Leon, 2020; Bushnaq et al. 2018; Ugwu, et al. 2020; Onah and Collins, 2020). Some of the recent studies of the HIV/AIDS models are presented below. Blower (Blower, 2012) shows that as more HIV-positive individuals gain access to treatment (HAART), incident rates of HIV fail. In their model, they assumed there will be a change of behavior by the treated HIV patients and thereby reducing their levels of exposure to the disease. Bachar and Dorfmayr (Bachar and Dorfmayr, 2004) show that treating individuals without reducing some of their dangerous conduct adds to the rise in the population of infected individuals.

It has been proven by recent studies (Anderson, et al. 1986; May and Anderson, 1987) that patients who get infected with HIV do not still change their behavior and soon might become infected again, even after knowing the risk that comes with the disease.

The models discussed above are mainly that of the integer-order differential equations (IDEs). But it is evident that many challenges that abound in different field of learning such as engineering, sciences, finance, economics, and in particular epidemiology can be better understood and analyzed by using fractional order differential equations (FDEs) (Khan et al., 2020; Abdo et al., 2020; Area et al., 2015; Farman et al., 2018). One of the properties of fractional-order models that stand it out from the integer-order is their non-local property which considers that what determines the future prediction of a model is not just a function of its current state, but it also depends on the historical antecedents of such models. This can be attributed to its ability to consider derivatives and integrals of arbitrary order (real and complex). Facts and truths in recent research are now better enhanced with the help of developing a fractional-order model, which makes it a way to go in developing and discussing both infectious diseases and other areas that deals with dynamical systems (Zafar et al., 2017; El-Saka, 2014; Sontakke and Shaikh, 2016; Shaikh et al., 2019; Nazir et al., 2020; Eiman et al., 2020; Taneco-Hernandez and Vargas-De-Leon, 2020). The past studies on infectious diseases and other related diseases have been studied using Riemann-Liouville fractional order derivative or the Caputo fractional order derivative, but these has been in recent times faulted where it shown that these derivatives pose a little challenge. Present studies have shown that at the end point of the interval where these models are defined their kernels have a singularity which creates discontinuity of the system at such point (Loh et al., 2018; Shah et al., 2020). As a result of these, many new definitions of fractional derivatives have now been proposed in the literature (Shah et al., 2020; Abdo et al., 2020; Shah et al., 2020; Toh et al., 2019; Kumar et al., 2020; Loh et al., 2018). The differences between different fractional derivatives are evident on the choice of kernels. The kernels are chosen such a way as to match with the required area of application. For instance, the main difference between the Caputo fractional derivative (Diethelm, 2010), the Caputo-Fabrizio derivative (May and Anderson, 1987), and the Atangana-Baleanu fractional derivative (Atangana, 2018) are that the Atagana-Baleanu fractional derivative is defined using a Mittag-Leffler law, the Caputo-Fabrizio fractional derivative is defined using an exponential decay law and the Caputo fractional derivative is defined using a power law. Various problems confronting man and nature have been addressed by computing an appropriate model, especially if these problems are dynamic. Most of these problems today are infectious diseases.

## Materials and Methods

To add to this current research area, we intend to develop the work previously done on HIV/AIDS by Cai et al, (Cai et al., 2009), where they subdivided the classes of HIV/AIDS transmission dynamics into four. These classes are S(t) the susceptible individuals, I(t) infected individuals in the asymptomatic phase, C(t) infected individuals who are in the symptomatic phase and A(t) denoting full-blown AIDS individuals. They also represented the total size of the population at the time t as N(t)=S(t)+I(t)+C(t)+A(t)

The classical order of HIV/AIDS, which is constructed by Cai et al, (Cai et al., 2009) is thereby given as


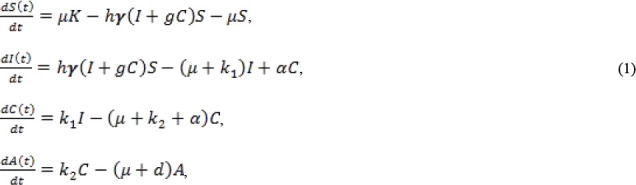


Where µK is the rate at which humans are brought into the population, µ is the natural death rate of humans, h is the mean number of contacts between human to human at each unit time, γ and gγ are probabilities that there is a disease transmission from an infective human in the asymptomatic stage and that of the symptomatic stage if they come in contact with a susceptible human respectively. k_1_ and k_2_ are the rates at which humans proceed from asymptomatic and symptomatic stages to the AIDS case, respectively. Also, α is the treatment rate of humans who are symptomatic so that they become asymptomatic and d is the death rate due to AIDS. The parameter unit can be taken in numbers of cells per cubic millimeters.

In this work, we advanced the work of Cai et al (Cai et al., 2009) by adopting the Caputo-Fabrizio operator of order β such that β ∈ [0, 1] as reflected in (May and Anderson, 1987; Shah et al., 2020). So we now rewrite (1) as


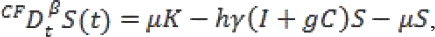
,


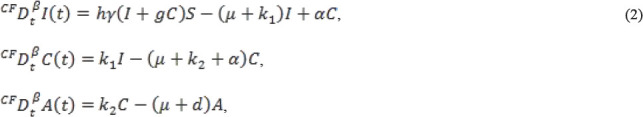


where 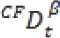 is the Caputo-Fabrizio fractional derivative, t>0. And with the initial conditions given below as


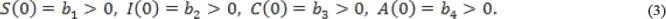


The classical model or integer-order model (1) is obtained when β=1. We will be using the iterative Laplace transform method to investigate the numerical outcomes of our model and compare with the classical case. In doing this we allocated arbitrary values to our parameters to verify our result.

For easy understanding, we arrange this paper as follows. In section 2, we presented some important definitions and remarks related to fractional derivatives. Section 3 was centered on obtaining the existence and uniqueness of our model equation. In Section 4, we obtained the equilibrium points of the model and showed that they are locally asymptotically stable under some conditions. We also analyzed the stability of our solutions using the iterative Laplace transform method of Picard successive approximation technique and fixed point theory accrued to Banach. Section 5 presents the numerical simulation of our result and finally, in section 6, we discussed our results and concluded.

### Preliminaries

**Definition 1.** (Caputo and Fabrizio, 2015) Let 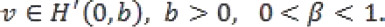 Then the fractional Caputo-Fabrizio differential operator is defined as:


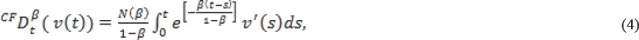


Where N(β) is a normalization function depending on β such that N(0)=N(1)=1. If the function fails to exist in H’(0,b), then the derivative can be redefined as


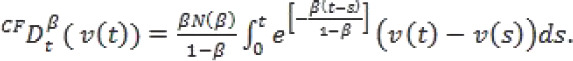


**Definition 2.** (Bashiri et al., 2018) Let 0<β<1, then the fractional Caputo-Fabrizio integral operator is given by


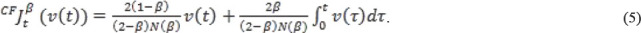


Just like in our usual Caputo derivative, if v is a constant function,
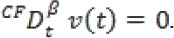
.

**Definition 3.** (Caputo and Fabrizio, 2015) Let 0<β<1, the Caputo-Fabrizio Laplace transform is given by


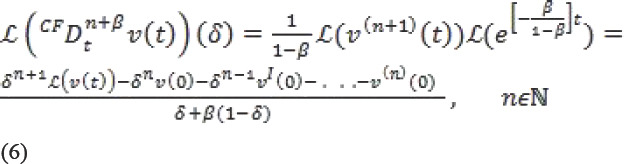


In particular, we have


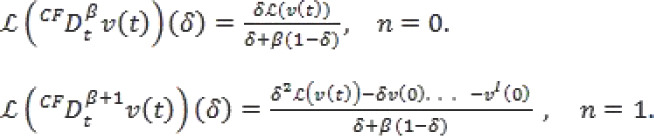


### Existence and Uniqueness

In this segment of our work, we examined the existence of a solution of the Caputo-Fabrizio fractional-order HIV/AIDS models (2) which were made possible with the help of Lemma 1, the fixed-point techniques (Kreszig, 1978; Hunter and Nachtergaele, 2001), and equation (5). Applying the Caputo-Fabrizio fractional integral operator (5) to both sides of equation (2), we have


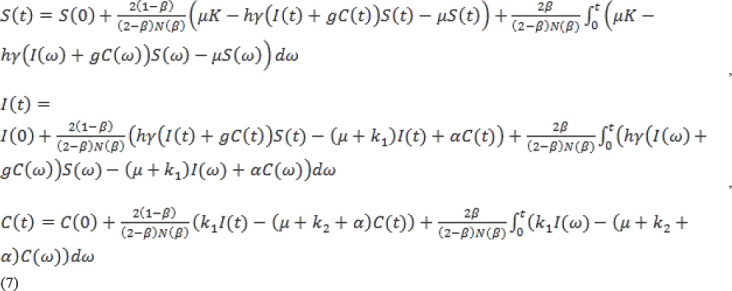
,


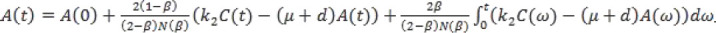
,

Let us consider the following kernels


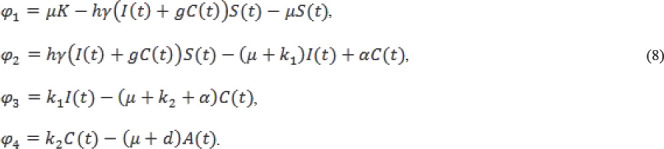
,

Let us take that *S, I, C, A* are bounded nonnegative functions with 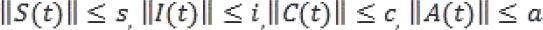 where *s,i,c* and *a* are some positive constants. Then we have


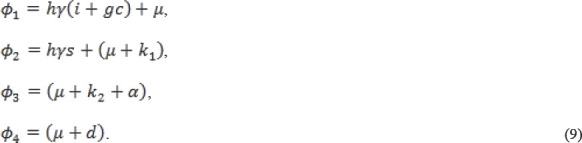
,

Rewriting the system given in equation (7) in terms of the kernel, we have:


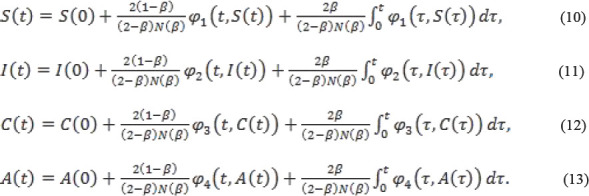


Using equation (10) – (13), we now state the following recursive formulas:


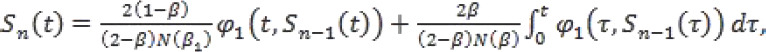



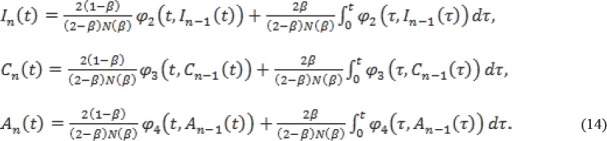


We proceed to consider the differences 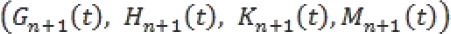 as follows:


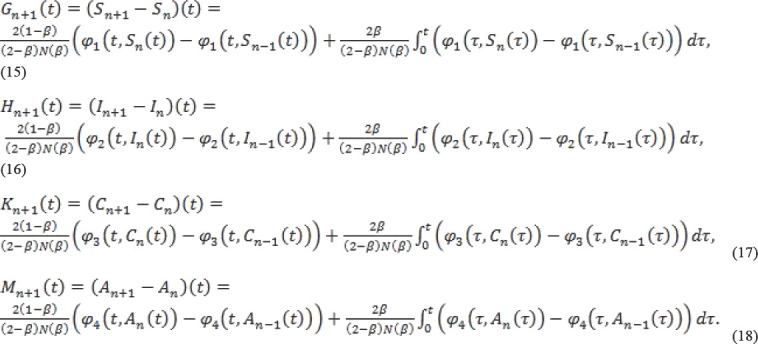


Note that:


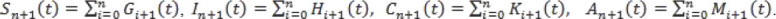
.

Taking the norm on both sides of equation (15) to (18) and by the triangular inequality for the differences 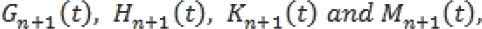 we have:


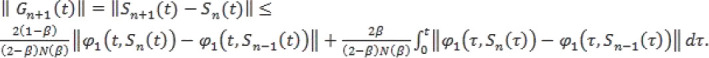


Given that the kernel φ_1_ satisfies the Lipschitz condition with associated Lipschitz constant φ_2_ (see appendix), we now have


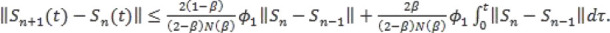


Thus, we obtain


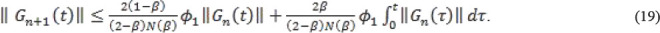


In similar way, the following results are obtained


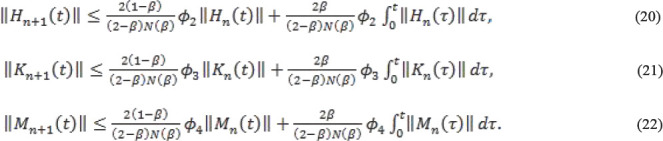


### Equilibrium points and Stability

In this section, we investigate the stability of model (2) by computing the equilibrium points of the model and thereby investigating the stability around those equilibrium points. The fractional-order system (2) has two equilibrium points. The disease-free equilibrium point (DFE), which is the equilibrium point when there is no HIV/AIDS in the system. Therefore, the DFE points of model (2) denoted by E^0^=(S^0^,I^0^,C^0^,A^0^) is obtained as

E^0^=(K,0,0,0)

To further study the dynamics of model (2), we consider a threshold quantity R_0_ called the basic reproduction number, calculated using the next-generation matrix approach of Van den Driessche and Watmough (Van den Driessche and Watmough, 2002). Applying this next-generation matrix approach, the basic reproduction number of model (2) in the absence of HIV/AIDS is obtained as


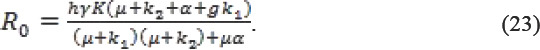


The quantity R_0_ above can be defined as a mean number of individuals that will be affected by HIV/AIDS if an infected human is introduced in the population. It is expected that when R_0_<0, HIV/AIDS will be eradicated from the human population in the absence of the introduction of more infected humans. On the contrary, if R_0_>0 then the disease will persist in the human population which may lead to a pandemic in the population.

Furthermore, the second equilibrium point is the endemic equilibrium points (EE), which is the equilibrium point when there is HIV/AIDS in the human population. Then the EE points of model (2) denotes by E^*^=(S^*^,I^*^,C^*^,A^*^) is also obtained as


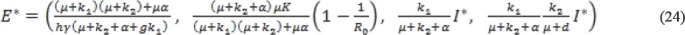


A dynamical system has both short- and long-term dynamics, the short-term dynamics is known by investigating the stability of the disease-free equilibrium, while the long-term dynamics is investigated by the stability of the endemic equilibrium (Onah et al., 2020). Consider the following fractional –order linear system described by the Caputo-Fabrizio derivative


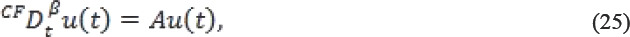


Where u(t) ∈R^n^, A∈R^nxn^ and 0<β<1.

**Definition 4.** (Bushnaq et al., 2018) The characteristic equation of the system (25) is 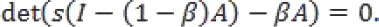


The linearization matrix of model (2) evaluated at the DFE point is


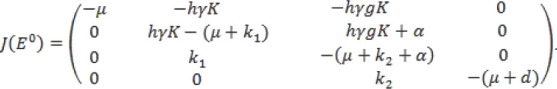
.

The characteristic equation of the linearized system of model (2) at E^0^ is


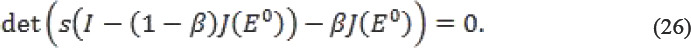


We can test the stability by applying iterative Laplace transform method on fractional HIV/AIDS model in which we obtain a stability for approximate solutions.

Consider model (2) having initial conditions as given in equation (3). Also, given two nonlinear terms in the model which are SC and SI. We proceed to apply the Laplace transform on both sides of (2), to get


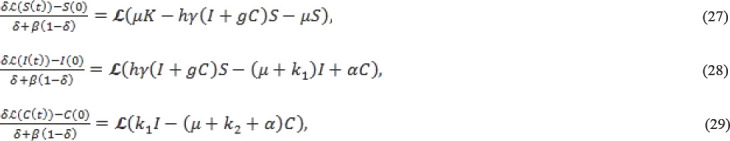



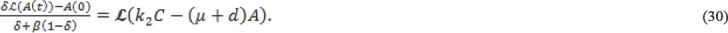


Rearranging, we have


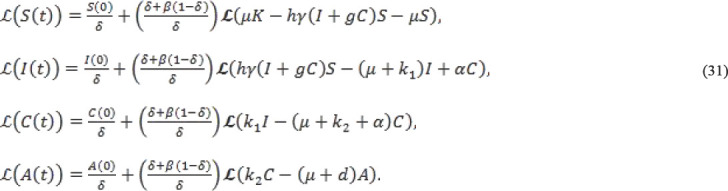


The inverse transforms of equations (31) yields


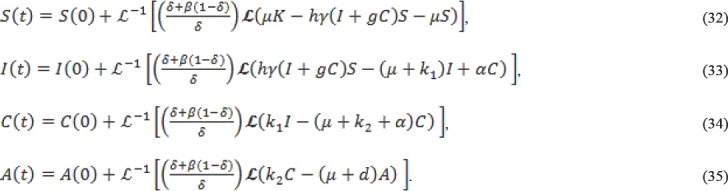


The infinite series solutions obtained by these methods given as,


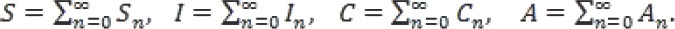


The nonlinearity SI and SC can be written as


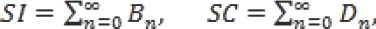


where B_n_ and D_n_ are written as follows


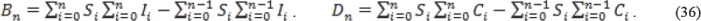


Next, we will obtain the following recursive formulas with initial condition (3)


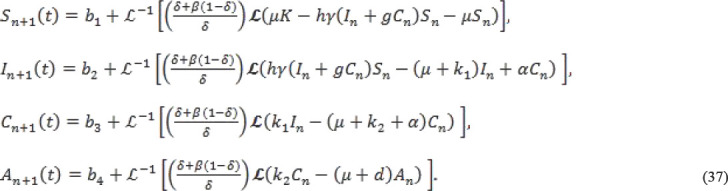


### Analysis of iterative method

Let (Y’,|| ·||) represent a Banach space and define Z as a self-map of Y’ Also, x_n+1_= ω(Z,x_n_) be a specific iterative scheme. Assume that F(Z) is a fixed-point set of Z possessing at least one element and that x_n_ converges to a point x∈F(Z). If {x_n_}⊆Y and define u_n_=||x_n+1=_ω(Z,x_n_)|| , and if lim_n->∞_ u^n^=0 implies that lim_n->∞_ x^n^=x then the iteration x_n+1_=ω(Z,x_n_) is said to be Z stable (Shaikh et al., 2019). (see theorems 5 and 6 in the appendix).

## Results

### Numerical Simulations

Recently, numerous analytical methods exist for solving various nonlinear fractional order models especially when the model deals with real life problems. Some of the new ones are as follows; Homotopy Analysis Transform Method (HATM) (Noeiaghdam and Ghiasi, 2017; Kumar et al., 2018), Local Fractional Homotopy Perturbation Laplace Transform Methods (LFHPLTM) (Singh et al., 2019; Sene and Fall, 2019), Homotopy Analysis Sumudu Transform Methods (HASTM) (Kumar et al., 2012; Alomari et al, 2020) and Adam-Bashforth-Moulton type predictor-corrector method (Diethelm et al., 2002; Pradeep et al., 2015). In this paper, we will use an iterative Laplace transform method (Shaikh et al., 2019) to obtain numerical solutions for Caputo-Fabrizio fractional model (2). The parameter values used for the numerical simulation are presented in Table 1 with some initial conditions 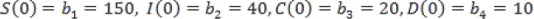 and considering a distinct value of the fractional order β=(0,1) . We present the underlisted graphical illustration to compare with intuitive prediction and analytical results.

**Table 1:** Parameter and assigned values from published data

Parameters	Parameter values	Source
*μ*	0.02	(Cai et al., 2009)
*K*	120	(Cai et al., 2009)
*h*	3	(Cai et al., 2009)
*γ*	0.0005	(Cai et al., 2009)
*g*	0.3	(Cai et al., 2009)
*k_1_*	0.01	(Cai et al., 2009)
*∝*	0.01	(Cai et al., 2009)
*k_2_*	0.85	(Cai et al., 2009)
*d*	0.04	Assumed

**Figure 1:**
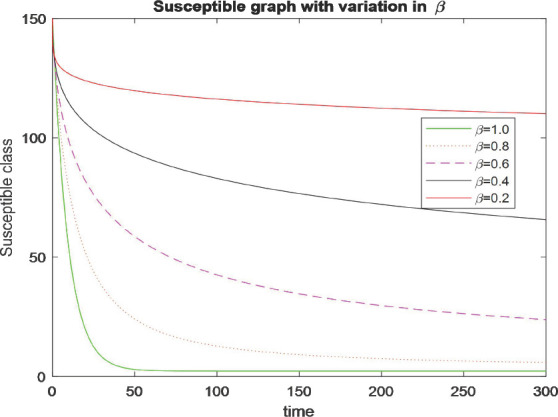
Graphical illustration of the susceptible class showing different values of β.

**Figure 2:**
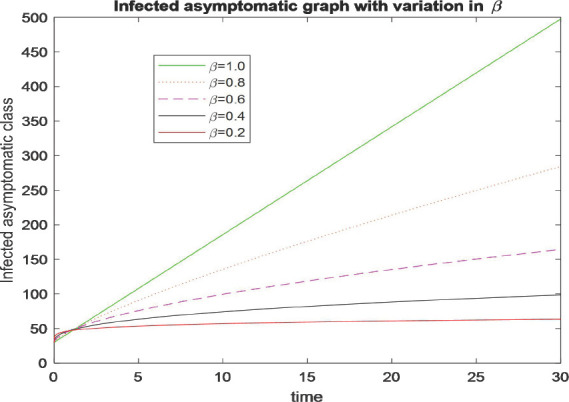
Graphical illustration of the infected asymptomatic class showing different values of β.

**Figure 3:**
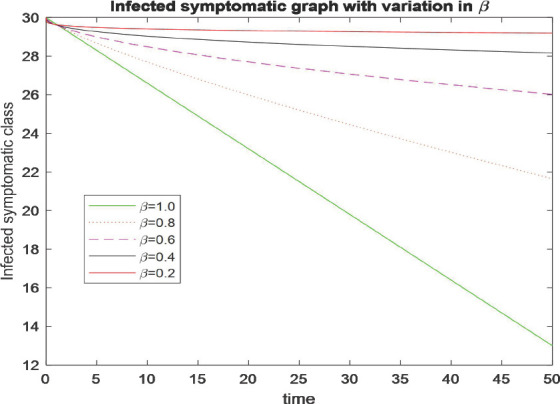
Graphical illustration of the infected symptomatic class showing different values of β.

**Figure 4:**
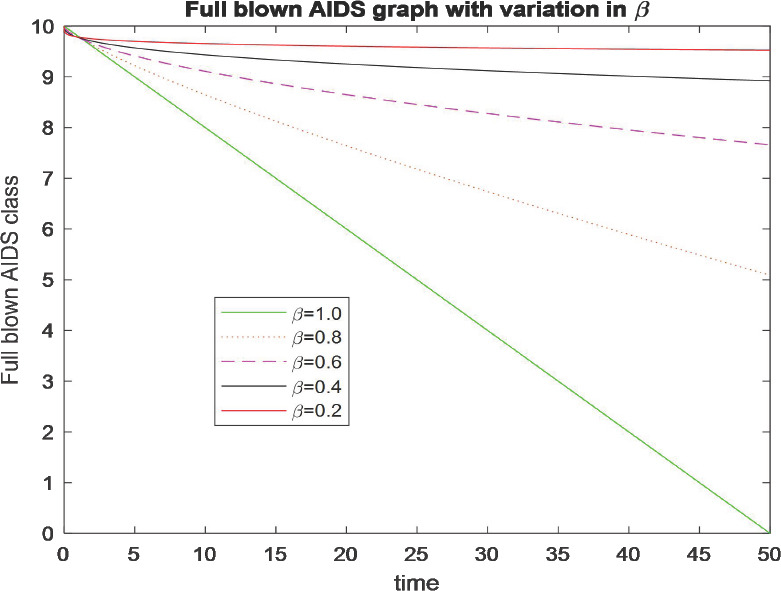
Graphical illustration of the full-blown AIDS class showing different values of β.

Figures 1 to 4 outlines the numerical simulations of our different model compartments, which are susceptible individuals S(t), infected asymptomatic individuals I(t), infected symptomatic individuals C(t) and individuals with full-blown AIDS A(t) for different values of β Here we employed the Iterative Laplace transform method (36) and (37) of fractional HIV/AIDS to explore the dynamics of this disease with the intent of comparing the fractional case with that of integer case. Using the above parameter values and β=0.2, 0.4, 0.6, 0.8 and 1.0, the roots of the characteristic equation (26) depending on the fractional order is solved numerically as follows. For β=0.2, the roots of Eq. (26) are 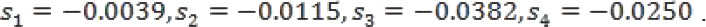 For β=0.4, the roots of Eq. (26) are 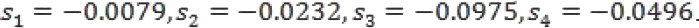 For β=0.6 , the roots of Eq. (26) are 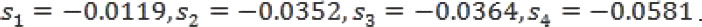 For β=0.8, the roots of Eq. (26) are 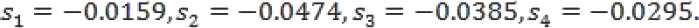 For β=1.0, the roots of Eq. (26) are 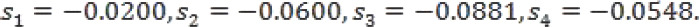 Hence, the equilibrium point E^0^ of model (2) is asymptotically stable for β=0.2, 0.4, 0.6, 0.8, 1.0 .

## Discussion

Figure 1 shows the dynamics of the susceptible individuals for β=0.2, 0.4, 0.6, 0.8 and 1.0 which is the classical case. We can see from the diagram that the susceptible class begins to reduce at β=0.2 and proceeds upwards. This implies that with fractional derivative, the effect of the disease on the susceptible class can be seen earlier than that of the classical case. From Figure 2 which is the infected asymptomatic individuals, it can be seen that the effect of the disease in the population is noticed earlier in the fractional case when compared to the classical case, as we can infer from the graph. In the infected symptomatic case, which is from Figure 3, there is decrease in the population of infectious symptomatic individuals as the equilibrium point and converges to non-zero for integer and non-integer value of β. These relations are also evident in Figure 4 respectively. From our figures, it is seen that HIV/AIDS begins to manifest earlier than reported in previous studies, but from our study it is seen that HIV/AIDS can be detected earlier using our model and method of analysis. This is helpful as earlier detection of this disease helps the individual in controlling and managing the disease to avoid escalating to a more horrendous level. It is also seen that as the fractional order ascends from β=0.2 to β=1, the population of HIV/AIDS infected humans becomes asymptotically zero. The result of our simulation also agrees with the analysis of Cai, et al (Cai et al., 2009) which is the classical case of our model as it is seen that when β=1 we get the same result as in Cai, et al (Cai et al., 2009) on their variation of the different variables. There is little difference in the two, which can be attributed to the different initial conditions chosen by the different authors, but our result validates the previous work done by (Cai et al., 2009) and shows that the use of fractional order derivative gives a quicker or faster evidence of the impact of HIV/AIDS on humans. Also, our study has been able to show and agree with the work of Cai et al. (Cai et al., 2009) that to reduce HIV/AIDS we need to reduce the reproduction number (R_0_) to be less than unity, so with our study early detection is guaranteed and this will enable epidemiologist to design better control strategies to limit rate of interaction between infected humans and uninfected humans and other possible preventive measures.

It is seen evidently that the numerical computation depends continuously on the time fractional derivative β. This shows that taken a given fractional operator like the Caputo-Fabrizio operator, it is less noisy and has a role in making a precise decision and thereby create opportunity (‘freedom’) to rewrite the model as to reflect data of specific patients, as it a better fit for the patients’ data. Meanwhile, the hybrid characteristic of Caputo-Fabrizio is adequate to enable the complex model and provide meaningful predictions. Epidemiologically, this means that with fractional operator, the disease will not become epidemic before its detected and relevant studies carried out to mitigate the disease. Also, it will pose less challenge to health practitioners in combating the disease. Our model does not consider control strategies for combating HIV/AIDS, but to show the advantage of using fractional order derivative in the analysis of the dynamics of HIV/AIDS over the classical case.

## Conclusion

In this paper, a Caputo Fabrizio fractional-order HIV/AIDS model has been investigated. The fractional derivative of fractional order β has been taken in the Caputo-Fabrizio sense which is a non-singular kernel. Further, by applying fixed point theory and an iterative method, the existence, uniqueness of the system of solutions for the model has been investigated and shown. The equilibrium points of the model were determined and the conditions for which the disease-free equilibrium point is locally asymptotically stable was also shown. An iterative Laplace transform method is used in obtaining numerical solutions of the fractional order system. We used the values of data used by (Cai et al., 2009), to create a good relationship with their former work done in the classical term. This work does not take care of prevention and control of HIV/AIDS but is looking at which possible and effective method in studying the dynamics of HIV/AIDS to give significant predictions of infection. Our simulations have helped in making a good comparison between the classical method and the fractional-order methods and given future epidemiologists a better and effective method in handling infectious disease epidemics.

### Conflicts of Interest

The authors declare that there is no conflict of interest regarding this study.

**List of Abbreviations:**
(HIV): Human immunodeficiency virus
(AIDS): Acquired immunodeficiency syndrome
(MAT): Medication assisted treatment
(WHO): World health organization
(HAART): HIV-positive individuals gain access to treatment
(FDE’s): Fractional order differential equations
(IDE’s): Integer order differential equations
(DFE): Disease-free equilibrium
(EE): Endemic equilibrium
(HATM): Homotopy Analysis Transform Method
(LFHPLTM): Local Fractional Homotopy Perturbation Laplace Transform Methods
(HASTM): Homotopy Analysis Sumudu Transform Methods

## Appendix

**APPENDIX**

**Lemma 1.** (Abdeljawad and Baleanu, 2016) The CF fractional integral and CF fractional derivative of the function v satisfies Newton-Leibnitz formular

**Lemma 2.** The kernels φ_1,_ φ_2,_ φ_3_ and φ_4_ in (8) satisfies the Lipschitz condition and are contraction mapping if

0< φ_1,_ φ_2,_ φ_3,_ φ_4<1._

**Proof.** Let S and S* be associated with kernel φ_1_, I and I^*^ be associated with kernel φ_2,_ C and C^*^ be associated with kernel φ_3_, A and A^*^ be associated with kernel φ_4_. Then,

Consider (38)

Consider (39)

Consider (40)

Consider (41)

Here ϕ_1,_ ϕ_2,_ ϕ_3_ and ϕ _4_ are defined in equation (8). Therefore, the Lipchitz conditions are satisfied for φ_1,_ φ_2,_ φ_3_ and φ_4._ In addition, since 0< ϕ_1,_ ϕ_2,_ ϕ_3,_ ϕ_4<1_ , the kernels are contractions.

**Theorem 1.** There exists a solution of fractional order HIV/AIDS model (2) provided that the following restriction holds.

**Proof:** Define

From equation (43)

Similarly

Using the same techniques, we have

Taking the limits on equations (47)- (50) as n-> ∞ and then using the condition (31), we have  We have now proved the existence of the system of solutions of system (2).

**Theorem 2.** (Uniqueness of Solution). A unique solution of the fractional order HIV/AIDS model (2) exists provided that (51) holds.

**Proof.** Let us assume an existing solution of system (2), like  satisfying the integral system given by equation (38) – (41), given as

Then

Taking the norm for both sides of equation (52) to (55) and applying lemma (2), we have

Form equation (56), we have

Using the same techniques, we have

By condition (51), we conclude that the inequalities in (60)- (63) are true, provided that  . This implies that . Thus, the model (2) has a unique solution.

**Theorem 3.** (Khan et al., 2020) The system (25) is asymptotically stable if and only if the real parts of the roots to the characteristic equation are negative and if (I-(1-β)A) is invertible.

**Theorem 4.** The disease-free equilibrium points E^0^ of model (2) is asymptotically stable if and only if real parts of the roots of the characteristic equation (26) are negative.

**Proof.** Given that equation (26) is a quartic polynomial equation, we expect four roots and hence denote the four roots by S_1,_ S_2,_ S_3_ and S_4_. The first two roots of equation (26) are trivial, which are given below as

.

It is obvious that S_1_ and S_2_ are negative since 0<β<1. We now determine the two remaining roots of equation (26) from the following equation.

where,

If the real parts of the two roots of equation (64) are negative, i.e Re(s_3_)<0 and Re(s_4_)<0 , then the equilibrium point E_0_ of model (2) is asymptotically stable by Theorem 3.

**Theorem 5.** (Shaikh et al., 2019). Let (Y’,|| ·||) be a Banach space and Z be a self-map of the Banach space Y’ satisfying

For all x,y ∈Y’ where 0 ≤M, 0 ≤m<1 Suppose that Z is a Picard Z -stable. Let us consider the equations (37) connected to (2).

Where  is a fractional Lagrange multiplier.

**Theorem 6.** let us consider a self-map Z which is defined as

Then the iterations are Z-stable in  if

**Proof.** We will show in our first step of proof that Z has a fixed point. Hence, we will evaluate the following for all (m,n ) ∈NxN

From equations (65)-(68), we take norm of the both sides of the equation without loss of generality, to get

Using the triangular inequality, (69) becomes

Since the obtained solutions play the same role, we suppose that

We now replace equation (70) with the approximation above, to obtain a new relation given as

Since I_n_, C_n_ and S_m_ are convergent sequences hence bounded, we can get three different positive constants σ_1,_ σ_2 and_ σ_3_ such that for all t,

By considering equation (71) and (72) we have

where f_1,_ f_2,_ f_3,_ f_4_ and f_5_ are functions emanating from  with

In the same procedure, we get

where d_1_, d_2,_ d_3,_ d_4,_ d_5,_ d_6,_g_1,_ g_2,_ h_1,_ h_2_ are functions emerging from  with  and  .

Therefore, the self-map Z has a fixed point. We are going to show next that Z satisfies the conditions associated with Theorem 5. Let (73)-(76) hold, then using m=0,

Thus, from equation (77), we can see that the mapping Z with conditions associated with Theorem 5 holds and are satisfied. Hence Z is Picard Z -stable.
